# The Days and Nights of Zoo Elephants: Using Epidemiology to Better Understand Stereotypic Behavior of African Elephants (*Loxodonta africana*) and Asian Elephants (*Elephas maximus*) in North American Zoos

**DOI:** 10.1371/journal.pone.0144276

**Published:** 2016-07-14

**Authors:** Brian J. Greco, Cheryl L. Meehan, Jen N. Hogan, Katherine A. Leighty, Jill Mellen, Georgia J. Mason, Joy A. Mench

**Affiliations:** 1 Center for Animal Welfare, University of California Davis, Davis, California, United States of America; 2 Department of Animal Science, University of California Davis, Davis, California, United States of America; 3 AWARE Institute, Portland, Oregon, United States of America; 4 Disney’s Animal Kingdom, Lake Buena Vista, Florida, United States of America; 5 Animal Science Department, University of Guelph, Guelph, Ontario, Canada; University of Tasmania, AUSTRALIA

## Abstract

Stereotypic behavior is an important indicator of compromised welfare. Zoo elephants are documented to perform stereotypic behavior, but the factors that contribute to performance have not been systematically assessed. We collected behavioral data on 89 elephants (47 African [*Loxodonta africana*], 42 Asian [*Elephas maximus*]) at 39 North American zoos during the summer and winter. Elephants were videoed for a median of 12 daytime hours per season. A subset of 32 elephants (19 African, 13 Asian) was also observed live for a median of 10.5 nighttime hours. Percentages of visible behavior scans were calculated from five minute instantaneous samples. Stereotypic behavior was the second most commonly performed behavior (after feeding), making up 15.5% of observations during the daytime and 24.8% at nighttime. Negative binomial regression models fitted with generalized estimating equations were used to determine which social, housing, management, life history, and demographic variables were associated with daytime and nighttime stereotypic behavior rates. Species was a significant risk factor in both models, with Asian elephants at greater risk (daytime: p<0.001, Risk Ratio = 4.087; nighttime: p<0.001, Risk Ratio = 8.015). For both species, spending time housed separately (*p<*0.001, *Risk Ratio* = 1.009), and having experienced inter-zoo transfers (*p<*0.001, *Risk Ratio* = 1.175), increased the risk of performing higher rates of stereotypy during the day, while spending more time with juvenile elephants (*p<*0.001, *Risk Ratio* = 0.985), and engaging with zoo staff reduced this risk (*p =* 0.018, *Risk Ratio* = 0.988). At night, spending more time in environments with both indoor and outdoor areas (*p =* 0.013, *Risk Ratio* = 0.987) and in larger social groups (*p =* 0.039, *Risk Ratio* = 0.752) corresponded with reduced risk of performing higher rates of stereotypy, while having experienced inter-zoo transfers (*p =* 0.033, *Risk Ratio* = 1.115) increased this risk. Overall, our results indicate that factors related to the social environment are most influential in predicting elephant stereotypic behavior rates.

## Introduction

Questions have been raised about whether zoos can adequately provide for the physical and psychological needs of elephants [1]. One concern is that zoo elephants perform abnormal behaviors (*e*.*g*., swaying, rocking, bobbing) that are stereotypic in nature for large portions of their time [2–4]. The development and performance of stereotypic behaviors in animals is associated with the inhibition of highly motivated behaviors, negative subjective states, and central nervous system dysfunction [5]. Additionally, animals who perform stereotypic behavior often live in environments that induce other signs of compromised welfare, such as physiological stress or health problems [4,5]. For these reasons, stereotypic behavior is considered one of the most important behavioral indicators of compromised welfare [5].

Several studies have provided information about the prevalence and rates of performance of stereotypic behaviors by elephants in a variety of settings (zoos: [3,6–10]; circuses: [11–14]; sanctuaries: [15]). These studies helped to identify variables within captive environments that contribute to elephant stereotypic behavior, but their generalizability is limited. Most had small sample sizes, and only three examined behavior at night, which is particularly important because nighttime for North American zoo elephants is, most commonly, 14 hours long [16,17]. Furthermore, only one [8] examined stereotypic behavior using multi-variable analyses. Multi-variable analyses allow for identification of multiple environmental and management factors that may interact to influence welfare outcomes, and thus are important for identifying areas for improvement [18,19].

Our study was designed to characterize the behavioral time budgets of 89 African (*Loxodonta africana*) and Asian (*Elephas maximus*) elephants from 39 North American zoos and apply multi-variable epidemiological methods to determine how management [16], housing [17], life history, and demographic [20] characteristics influence stereotypic behavior rates. Epidemiology examines patterns of outcomes in populations to understand risks, and is increasingly being applied to assessments of animal welfare in agricultural and zoological settings [18,21]. Building epidemiological models relies on an iterative process involving the testing and winnowing down of hypothesis-supported input variables. We tested 42 such input variables to determine their relationships to stereotypic behavior rates (i.e., the proportion of time spent daily performing stereotypic behavior), which is an indicator of the severity of stereotypic behavior performance. In the remainder of the introduction we discuss the background for some of the key hypotheses; further information about the input variables we evaluated and their hypothesized direction of effect is provided in Table 1.

**Table 1:** Independent variables tested for association with stereotypic behavior rates.

Variable Class	Hyp^1^	Variable	Unit of Analysis	Unit	Description
	-	Animal Contact	Elephant		Maximum number of unique elephants focal animal is in contact with
	-	Herd Size	Zoo		Total number of elephants at zoo
	+	Percent Time Housed Separately	Elephant	%	Percent time spent housed in a social group of one
	-	Percent Time Mixed Age Group	Elephant	%	Percent time spent in social groups composed of individuals from at least two different age classes
	+	Percent Time Single Age Group	Elephant	%	Percent time spent in social groups composed of individuals from the same age class
Social^2^	+	Percent Time Single Sex Group	Elephant	%	Percent time spent in social groups composed of individuals of the same sex
	-	Percent Time w/ Juveniles	Elephant	%	Percent time spent in social groups where an elephant seven years or younger was present
	+	Relative Social Experience Change	Elephant		(Total daytime Social Experience—Total nighttime Social Experience)/(Total daytime Social Experience)
	-	Social Group Contact	Elephant		Maximum number of unique social groups focal animal is part of
	-	Social Experience	Elephant		The average weighted (by percent time) size of all social groups in which an elephant spent time
	-	Environment Contact	Elephant		Maximum number of unique environments an elephant was housed in
	+	Percent Indoor	Elephant	%	Percent time spent in indoor environments
	-	Percent In/Out Choice	Elephant	%	Percent time spent in environments with an indoor/outdoor choice
	-	Percent Outdoor	Elephant	%	Percent time spent in outdoor environments
	+	Percent Time on Hard Substrate	Elephant	%	Percent time spent in environment with 100% concrete or stone aggregate substrate
	-	Percent Time on Dirt Substrate	Elephant	%	Percent time spent in environment with 100% dirt substrate
	-	Percent Time on Soft Substrate	Elephant	%	Percent time spent in environment with 100% grass, sand, or rubber substrate
Housing^2^	-	Percent Time on Soft Substrate or Dirt	Elephant	%	Percent time spent in environment with 100% grass, sand, rubber, or dirt substrate
	+	Relative Space Experience Change	Elephant		(Total Day Space Experience—Total Night Space Experience)/(Total Day Space Experience)
	-	Space Experience	Elephant	ft^2^	The average weighted (by percent time) size of all environments in which an elephant spent time
	-	Space Experience Per Elephant	Elephant	ft^2^	The average weighted (by percent time) size of all environments in which an elephant spent time divided by the number of elephants sharing the environment
	-	Space Experience Indoor	Elephant	ft^2^	Average size of the environment an elephant spends time in weighted by the amount of time spent in that environment (for indoor environments only)
	-	Space Experience Outdoor	Elephant	ft^2^	Average size of the environment an elephant spends time in weighted by the amount of time spent in that environment (for outdoor environments only
	-	Enrichment Diversity	Zoo		Shannon diversity index of the number of enrichment types and frequency with which they were provided. Higher scores indicate more divers experience of enrichment types
	-	Enrichment Program	Zoo		Standardized Factor Score describing the frequency of use of the different components of an enrichment program. Enrichment programs with higher scores are more rigorous and scientifically based.
	-	Exercise Per Week: (less thanone hour per week)	Elephant		Number of reported hours spent exercising animals each week
	-	Feed (Day or Night)	Zoo		The number of feedings during the daytime or nighttime
Management^3^	-	Feeding Predictability	Zoo		The predictability of feeding activities, ranging from predictable to unpredictable
	-	Alternate Feeding Methods	Zoo		Relative frequency of the percentage of time food was presented in a foraging device, hidden, or hanging compared to all feeding types
	+	Percent Guide Interaction Time (0% of interactions involve a guide)	Elephant		Percentage of time and elephant spent engaged with or overseen by trainers who had a guide on their person
	-	Percent Time Managed	Elephant	%	Sum of percent time spent in managed activities
	-	Rewarding Stimuli Techniques Score (Sometimes/frequent experience)	Elephant		Percent time with which an elephant experienced techniques involving the provision or removal of rewarding stimuli divided by the percent time all training techniques were experienced.
	-	Walk Week: (less than one hour per week)	Elephant		Number of reported hours spent walking animals each week
	-	Experience Birth	Elephant		Elephant was at a facility when a calf was born
	+	Experience Death	Elephant		Elephant was at a facility when an elephant death occurred
Life History^4^	-	Separation Age	Elephant		Age at which an elephant was separated from his/her mother
	-	Separation by Age	Elephant		Separation age divided by current age
	+	Transfers	Elephant		Total number of inter-zoo transfers and elephant has experienced
	+	Age	Elephant		Age of elephant (years)
Demographic^4^	-	Origin: (Imported from home-range)	Elephant		Captive born or imported from home-range
		Sex: (male)	Elephant		Male or Female
		Species: (African)	Elephant		African or Asian
Other	NA	Season: (winter)			Winter or summer

^1^ Hypothesized direction of effect

^2^ Detailed descriptions of Social and Housing variables can be found in Meehan et al. [17].

^3^ Detailed descriptions of Management variables can be found in Greco et al. [16].

^4^ Detailed descriptions of Life History and Demographic variables can be found in Prado-Oviedo et al. [20].

### Social, Housing, and Management Variables:

There is evidence suggesting that the social and spatial experiences of captive elephants [11,15,22] and other animals [5] influence stereotypic behavior performance. Raising and housing animals in environments that lack variability, complexity, or novelty can reduce opportunities for behavioral expression, cause frustration because the animals cannot engage in highly motivated behaviors, and lead to prolonged negative subjective states [5]. Such environments may even alter processing in the brain’s cortical-basal ganglia systems in a way that promotes stereotypic behavior [5]. Thus, we hypothesized that social and spatial variables likely to indicate a sub-optimal environment or that would potentially constitute sources of frustration (e.g., social separation, spatial restriction) would correspond with increased stereotypic behavior risk. Conversely, we hypothesized that social and spatial variables reflecting greater environmental complexity and more opportunities for the elephants to perform highly motivated behaviors (e.g., more time spent with herd mates, opportunities to choose between different housing areas) would correspond with reduced risk.

Unlike the effects of social and spatial experiences, there is not much diversity in studies examining the effect of management practices on stereotypic behavior. Some management practices, like positive reinforcement based training, environmental enrichment, and unpredictable feeding schedules, have been repeatedly demonstrated to reduce stereotypic behavior in many non-elephant species [23–27]. Such practices are believed to reduce stereotypic behavior because they increase environmental complexity, facilitate behavioral expression, and buffer stress [23–27]. Thus, we hypothesized that increased access to a variety of types of environmental enrichment, positive reinforcement based training, and feeding elephants on unpredictable schedules would correspond with reduced stereotypic behavior risk. Other management practices, like staff directed exercise, time managed, and the use of guides (ankuses) have not been investigated, but we hypothesized that they could also influence stereotypic behavior.

### Life History Variables

There are a variety of life history events that contribute to stereotypic behavior. For example, early weaning and/or maternal separation are reported to be risk factors for stereotypic behavior in a wide variety of animals, including cats, cattle, chickens, horses, laboratory mice, mink, primates, rats, swine (as reviewed by Novak et al. [28]) and elephants[15]. This risk likely stems from the disruptions to social groups and interrupted behavioral development [28]. Additionally, experiencing inter-zoo transfers or the death of a herdmate can disrupt social relationships and may occur repeatedly throughout an elephant’s life time. Thus, we hypothesized that younger ages of maternal separation and increased experiences with transfers or deaths of herd mates would increase stereotypic behavior performance rate risk.

### Demographic Variables

Age and birth origin often predicts stereotypic behavior. For elephants and several other species, stereotypic behavior has been shown to increase in frequency with age (elephants: [8,15], mice: [29], bears: [30]). We therefore hypothesized that older zoo elephants would perform stereotypic behavior at higher rates than their younger conspecifics. Evidence from studies of primates, rodents, and carnivores also shows that wild-captured animals are less likely to develop stereotypic behavior than their captive-born counterparts, suggesting that maturation in the wild can have protective effects [31]. The studbooks we used to collect life history data only characterize elephants as either American/European captive-born or imported from home-range countries [20]. With the understanding that some elephants imported from their home-range countries might not have been wild-captured, we hypothesized that captive-born elephants would be more prone to performing stereotypic behavior at higher rates than their imported counterparts.

Species and sex effects on stereotypic behavior are more nuanced than those associated with age and birth origin. Although there are well-documented differences in stereotypic behavior between phylogenetically different species (e.g., ruminants are prone to develop oral stereotypic behaviors while carnivores are prone to develop locomotor stereotypic behaviors like pacing [5,32]), differences in stereotypic behavior performance for phylogenetically similar species have only been investigated in a limited number of studies [5,33]. However, there is some evidence that Asian elephants are more at risk than African elephants [4]. There is no evidence to suggest that sex influences the stereotypic behaviors of elephants. Some species of animals consistently demonstrate sex effects (higher rates in males: [34]; higher rates in females [35]), while others do not [36]. Therefore, we hypothesized that there might be previously undetected sex effects on the stereotypic behaviors of elephants.

## Methods

### Ethics statement

The management at each participating zoo authorized this study and the Zoological Society of San Diego’s Institutional Animal Care and Use Committee approved the study protocol on behalf of all participants (N.I.H. Assurance A3675-01; Protocol 11–203). Our study was non-invasive.

### Data Collection

We collected video data from 89 elephants (Africans: N = 7 males, 40 females; Asians: N = 8 males, 34 females) housed at 39 Association of Zoos and Aquariums accredited institutions. Requests to participate in the project were sent to 68 zoos, of which 39 decided to participate. Of the 39 zoos, 22 housed two females and 17 housed more than two females. To normalize representation across zoos, we blindly selected (using randomized identification numbers) two female elephants who were older than 12 years and free from chronic illnesses. Fifteen of the 39 zoos housed males that met the same age and health criteria. Nine zoos had one male and six had more than one male. We blindly selected one male from each of these 15 zoos using the same process as was used to select the female participants.

Daytime (regular zoo operating hours) data collection for this project took place during winter (December to February) and summer (June to August) 2012. Non-elephant care staff at each zoo used Bell and Howell DV800HD Hi-Definition Camcorders to collect video data from all elephants. Before the data were collected, we worked with each zoo to develop individualized video recording schedules. Elephant managers reported the times of day during which the elephants were either engaged in managed activities or had independent time (time spent outside of staff-directed activities) [16]. We used the independent time information to assign weekly observation blocks evenly distributed between the morning and afternoon. Staff members were encouraged to collect videos at different times during both the morning and afternoon time blocks to capture variability in behavioral performance. Staff collected one full hour of video every session. However, no more than one hour of video was recorded per week per elephant, and only one elephant could be recorded on any specific day. The video recording protocol required the collection of 12 hours of video per elephant per season, although we received between 8 and 12 hours (hours of video collected per elephant: winter: median = 12, IQR = 0; summer: median = 12, IQR = 1; combined: median = 24, IQR = 1). We excluded any elephant with fewer than 10 hours of video per season from the data coding process.

The nighttime (zoo non-operating hours) data collection took place in the fall (September-November) of 2012. We used portable night-vision equipment (Bushnell 6x50mm Equinox night vision monocular and Sight Mark 3.5x42mm Twilight digital night vision monocular with a Streamlight TL-2 IR spotlight) to observe a subset of 32 elephants (Africans N = 3 males, 16 females; Asians N = 3 males, 10 females) from 14 of the participating zoos. Twenty minutes of data were collected during each hour of the night. The range of hours of data collected during night time observations was between 5.3 and 16 hours (median = 10.5, IQR = 2.3).

Daytime behaviors were coded from digital video recordings by observers trained to meet 85% inter and intra observer reliability criteria. Observer ratings were spot-checked monthly to ensure that the 85% reliability criterion was maintained throughout the behavior coding process. Nighttime behaviors were coded during live observations by a single observer (BG) who also trained using the daytime video recordings to meet the 85% reliability criteria. We used instantaneous sampling at five minute time intervals to code all behaviors. At each time interval, the focal elephant was scored as being non-visible, engaging in resting behavior, or performing one of the following eight active behaviors: stereotypic behavior, feeding, locomotion, feeding while locomoting, self-maintenance, social contact, object manipulation, or other (see Table 2 for definitions).

**Table 2:** Ethogram.

Behavior Type*	Definition
**Feeding**	Picking up, manipulating, and/or consuming food and/or water.
**Locomotion**	Walking or running more than two body lengths.
**Feeding while Locomoting**	Engaging simultaneously in “feeding” and “locomotion.”
**Rest**	Standing or lying without engaging in another listed behavior.
**Self-maintenance**	Tossing friable or liquid substances onto the body; rubbing the body on object and/or partially or fully submerging the body in water or mud.
**Social Contact**	Physically contacting one or more conspecifics in a social context. Social contact included social greetings, play, reproductive behavior, and aggression. Inadvertent touches attributable to ear flapping, tail swishing, stereotypic behavior, or shifting of body weight were excluded.
**Stereotypic Behavior**	Performing motor, locomotor, oral, and/or self-directed behavior for three or more repetitions without interruption.
**Object Manipulation**	Manipulating non-food items with the trunk and/or foot. Interactions with enrichment items that distribute food were included as long as the primary behavior was directed towards the object and not the food.
**Other**	Interacting with guests or other non-elephant animals

*All behavior types except rest were considered to be active behaviors

### Data Processing and Analyses

The daytime and nighttime behavior time budgets were calculated by dividing the counts of each behavior by the total number of active visible samples (not resting) from each observation period. This method corrects for individual differences in general activity [29,37]. Since the data were count-based proportions and often non-normally distributed, descriptive statistics were calculated and Mann-Whitney U (Wilcoxon Rank Sum) tests were used to test for species, sex, and period effects (daytime/nighttime) within each behavioral category. We did not test for sex effects in the nighttime data because there were too few males to support robust analyses. Microsoft Excel (Microsoft Corp, Redmond, WA) and/or SAS v.9.3 (SAS Institute, Cary, NC) were used for all descriptive and statistical calculations. A *p*-value of <0.05 was considered statistically significant.

Prior to conducting analyses we developed a data set in which the winter stereotypic behavior observations were paired with housing and social values collected during January, and summer stereotypic behavior observations with housing and social values collected during July. It was not possible to match management values to the winter and summer observations because management values were calculated from a survey characterizing year-round practice [16]. Once the data set was established, we screened all daytime and nighttime independent variables for outliers. For continuous variables, outliers were removed if they were three standard deviations away from the mean, while count-based outliers were removed if they exhibited a Cook’s D value greater than 4/N [38]. For categorical risk factors, we excluded any category with fewer than 10 individual observations from further analyses.

Both daytime and nighttime stereotypic behavior rates were investigated using epidemiological models. Negative binomial regression was first used to verify whether daytime stereotypic behavior rates were consistent across seasons and from day to night. Following these analyses, separate daytime and nighttime negative binomial regression models were used to determine which individual risk factors were associated with stereotypic behavior rates. These predictive models were fitted using generalized estimating equations (GEE), which allow for the individual elephant to be used as the unit of analysis, account for clustering of individuals within zoos, and support repeated measurement of individual elephants [39,40]. Zoos were treated as random effects and an independent correlation structure was specified [41]. Residual over-dispersion was adjusted for using a Pearson deviance scale [42]. Multi-variable regression models were built by first assessing individual predictors at the univariate level and then at the bivariate level with demographic variables determined to be potential confounders (age, sex, species, season, and origin)[43,44]. Confounding variables (those demographic variables that affected stereotypic behavior rate and altered the beta values of input variables by more than 10% during bivariate analysis) were included in all models, and any variables that predicted stereotypy rates (*p<*0.15) following the univariate or bivariate assessments were retained for evaluation in the hierarchical model building process.

Once a pool of viable input variables and confounders was identified, the hierarchical model building process proceeded using the forward selection approach [45]. Models reaching the multi-collinearity criteria, as defined by a variance inflation factor of greater than 10 and a condition index of greater than 30, were not considered for further analysis [45]. The forward selection of variables was continued until the addition of variables no longer resulted in significant models. Interactions were assessed during the final model building stage and the final model was selected based on quasi-likelihood under the independence model criterion (QIC) values [46] and parameter estimates of explanatory variables. Risk ratios were calculated to quantify the effect input variables had on the probability of outcomes. Since negative binomial regression models are based on the logarithmic scale, risk ratios (RR) for stereotypic behavior rates were calculated by exponentiation of the calculated beta-coefficients [47]. Statistical analyses were conducted by using SAS software, version 9.3 [PROC GENMOD, with options DIST = NEGBIN, LINK = log, SCALE = P, TYPE = ind, and REPEATED; SAS Institute, Inc., Cary, NC]. With the exception of the univariate stage of the model building process where *p*<0.15 was considered acceptable for continued analyses, *p*<0.05 was considered statistically significant in the remainder of the model building stages.

Similar, although not identical, sets of input variables were tested in the daytime and nighttime models. Some variables were specific to time of day (*e*.*g*., management activities never occur at night), and other variables were insufficiently variable to test in both models (*e*.*g*., only 6 of the 32 subjects in the nighttime population spent time with young elephants during the night). All variables evaluated, along with their hypothesized directions of effect, are shown in Table 1. To keep the results and discussion concise, we have used the variable names (capitalized) defined in Table 1 and more fully described in Greco et al. [16], Meehan et al. [17], and Prado-Oviedo et al. [20].

Following the model building process we sought to determine whether our population was representative of the full adult elephant population (N = 217). To do so, we use the least squared means procedure to compare the means of significant independent variables from the daytime and nighttime models to the means on the same variables for the full adult elephant population [16,17,19,20].

## Results

### Behavioral Time Budgets

**Fig 1:**
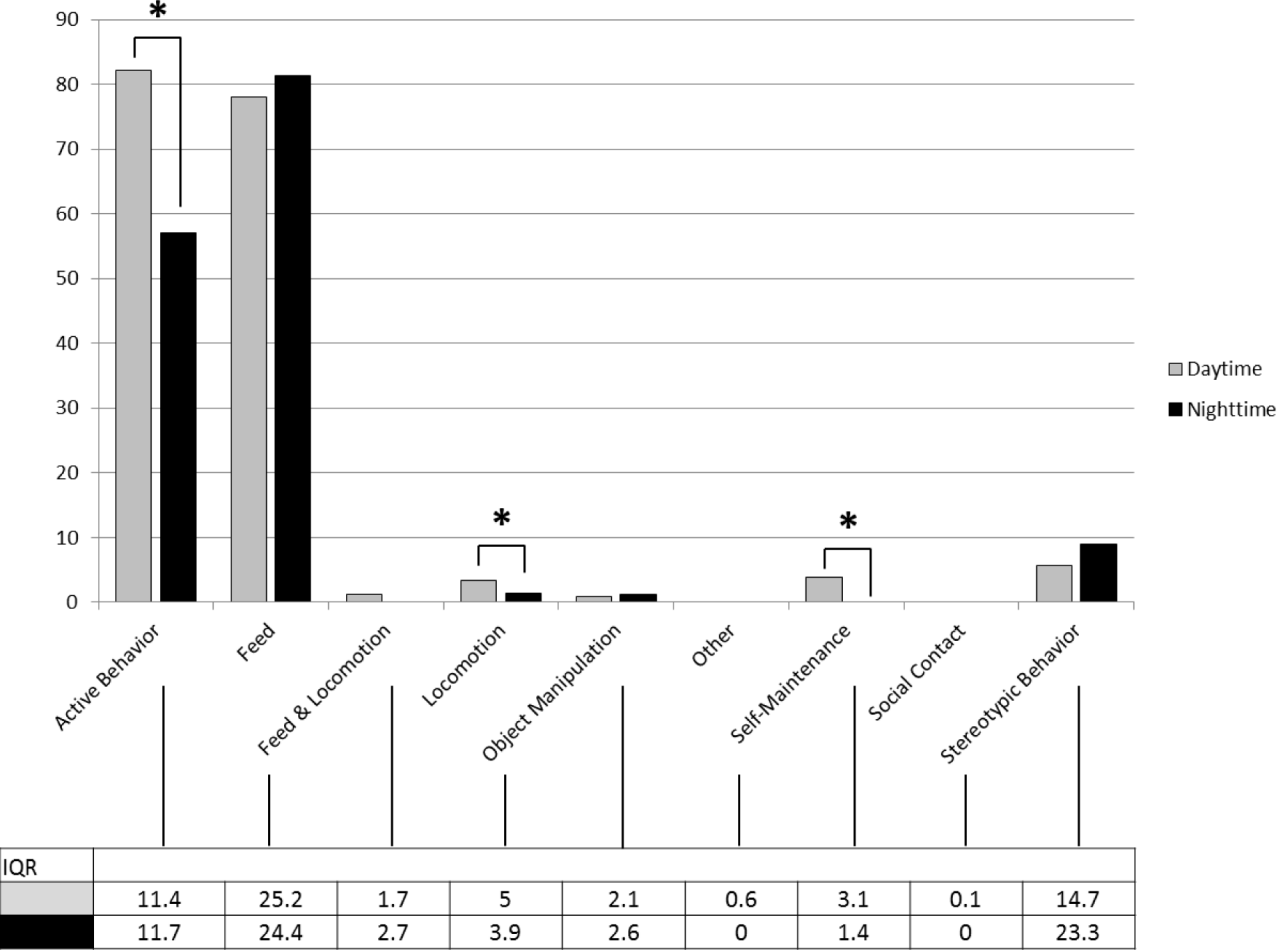
Median Day and Nighttime active behavior percentages. Asterisks represent significant period differences (p<0.05). In the Daytime and Nighttime, median values for Other and Social Contact were zero. The median value for Feed & Locomotion was also zero during the Nighttime.

The elephants were active for approximately 80% of daytime observations and 60% of nighttime observations. The most common active behaviors during both the day and the night were feeding and stereotypic behavior. The elephants were more active overall during the daytime than the nighttime (Fig 1), particularly with respect to locomotion and performing self-maintenance behavior.

**Fig 2:**
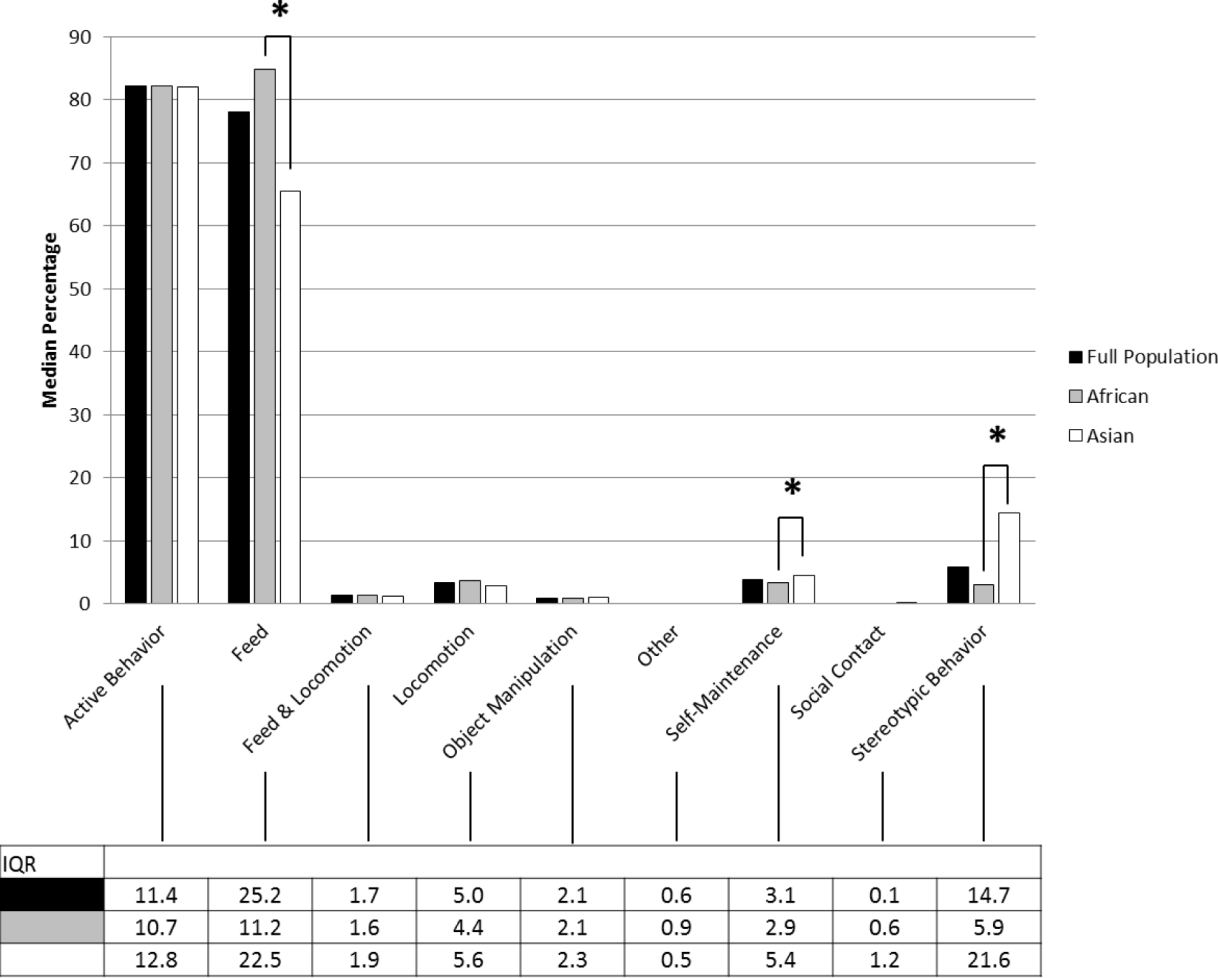
Median daytime active behavioral observation percentages and interquartile ranges. Asterisks represent significant species differences (p<0.05). For the full population, African and Asian elephants, the median values for Other were zero. The median values for Social Contact were also zero in full population and African elephants.

**Fig 3:**
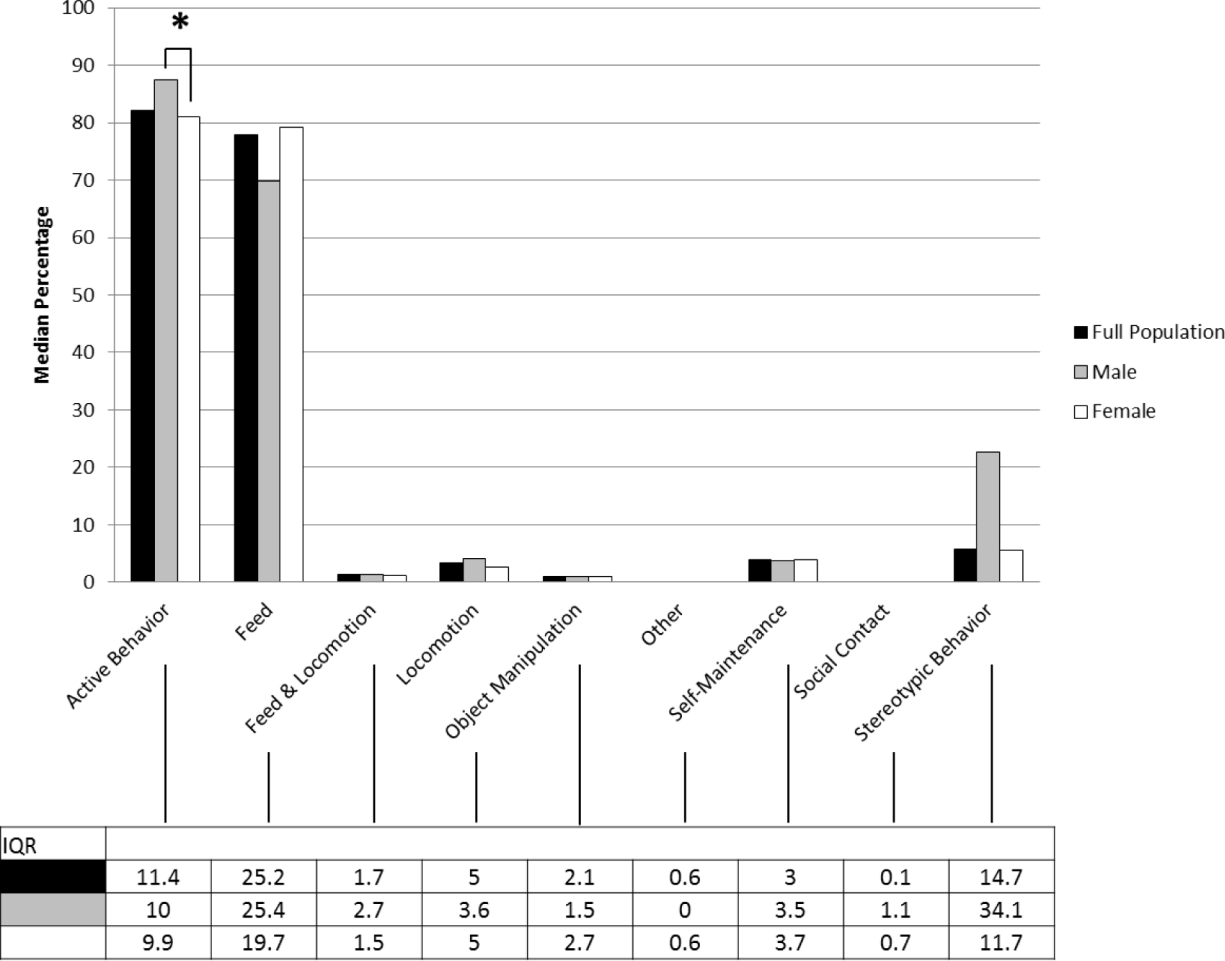
Median daytime active behavioral observation percentages and interquartile ranges. Asterisks represent significant sex differences (p<0.05). For the full population, males and females, the median values for Other and Social Contact were zero.

**Fig 4:**
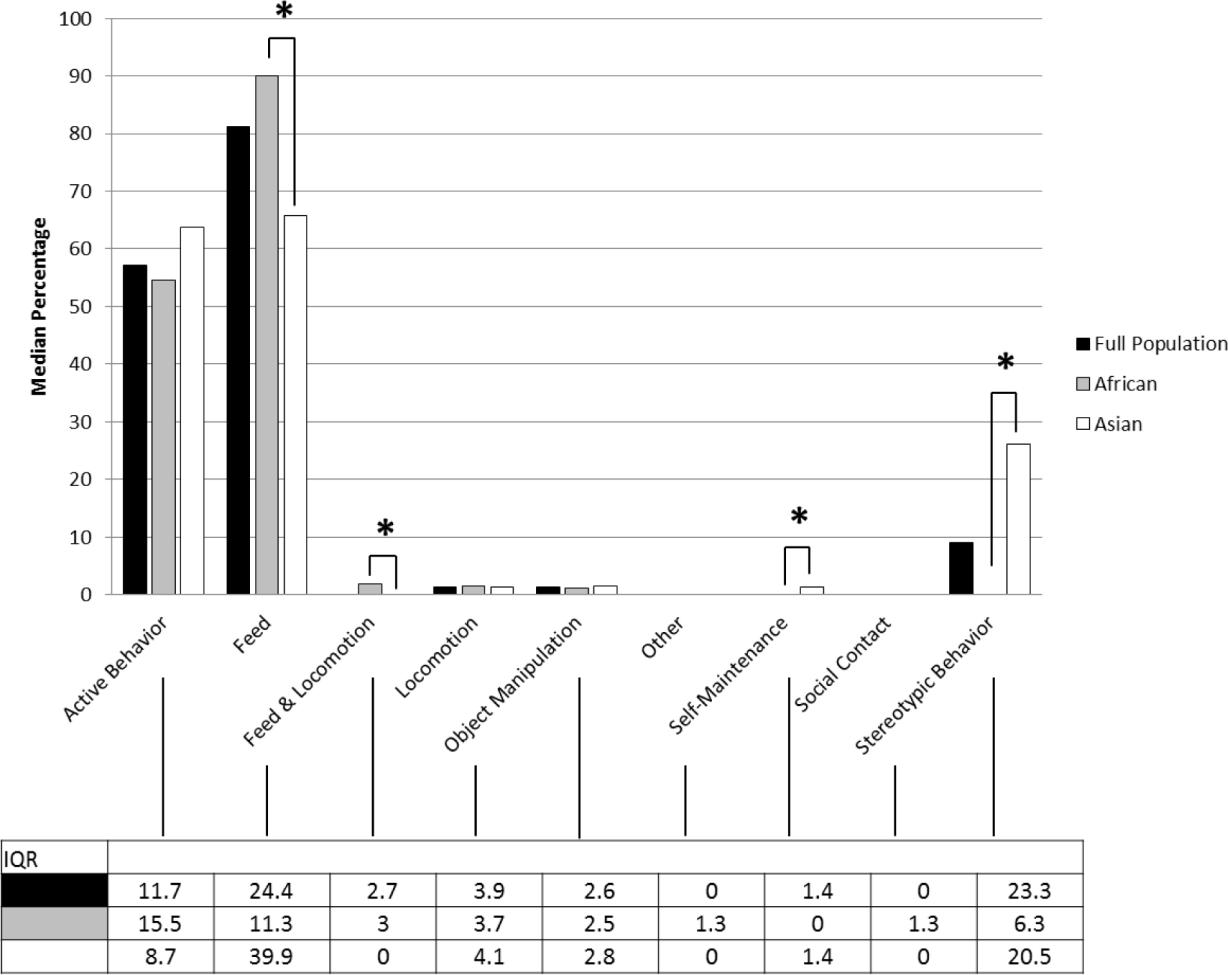
Median nighttime active behavioral observation percentages and interquartile ranges. Asterisks represent significant species differences (p<0.05). For the full population, median values for Feeding & Locomotion, Other, Self-Maintenance, and Social Contact were zero. For African elephants, median values for Other, Self-Maintenance, Social Contact, and Stereotypic Behavior were zero. For African elephants, median values for Feeding & Locomotion, Other, and Social Contact were zero.

African elephants fed for a significantly greater proportion of observations than Asians and Asian elephants performed self-maintenance and stereotypic behavior for a greater proportion of observations than Africans (Fig 2). Male elephants were generally more active than female elephants during the daytime (Fig 3). During the nighttime, African elephants fed and fed while locomoting for a significantly greater proportion of observations than Asians, and Asians performed self-maintenance and stereotypic behavior for a greater proportion of observations than Africans (Fig 4).

### Stereotypic Behavior

**Fig 5:**
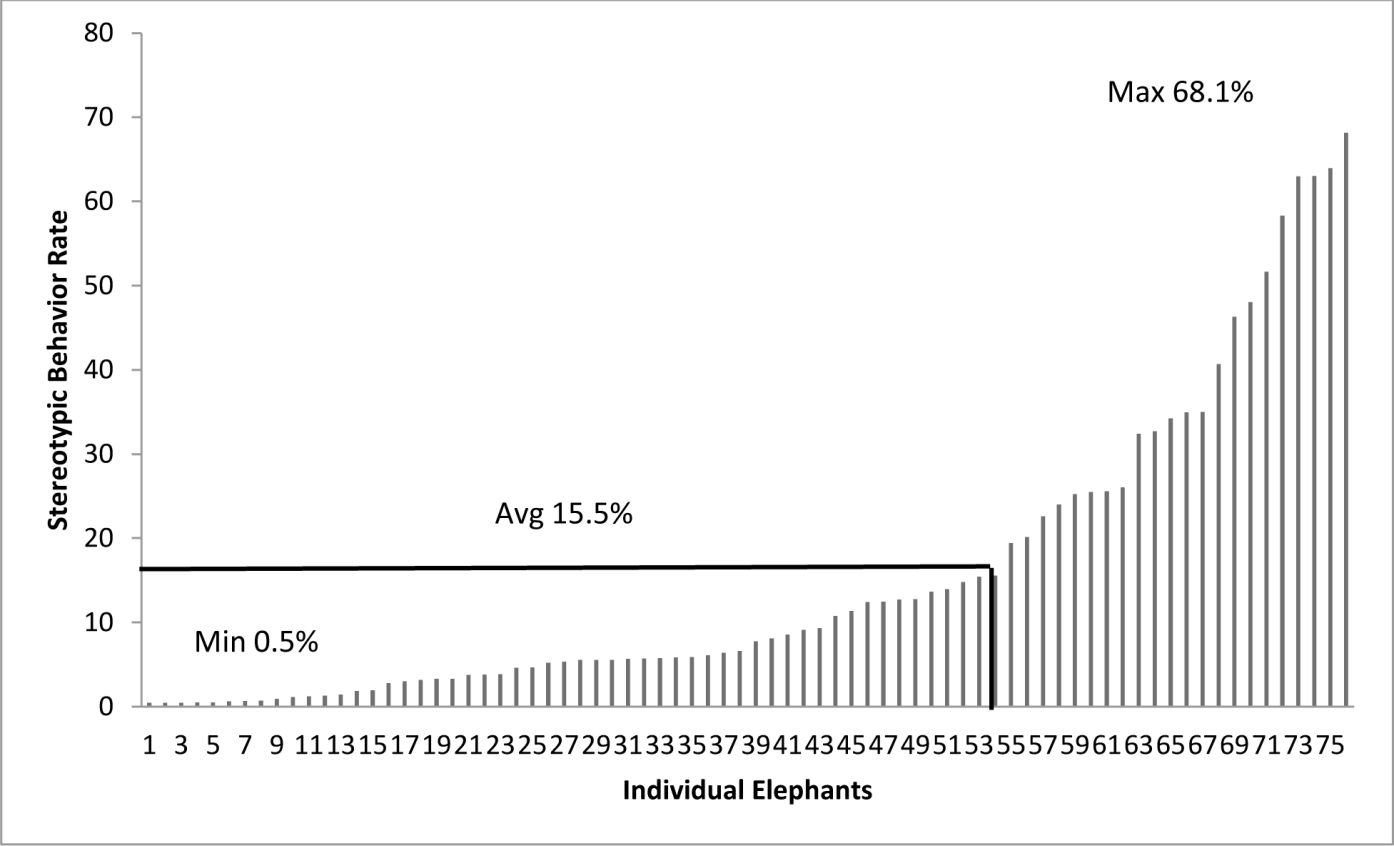
Daytime stereotypic behavior performance rates calculated as a percentage of active time for individual elephants. The average stereotypic behavior rate in the full population was 15.5%, the min 0.5%, and the max 68.1%.

**Fig 6:**
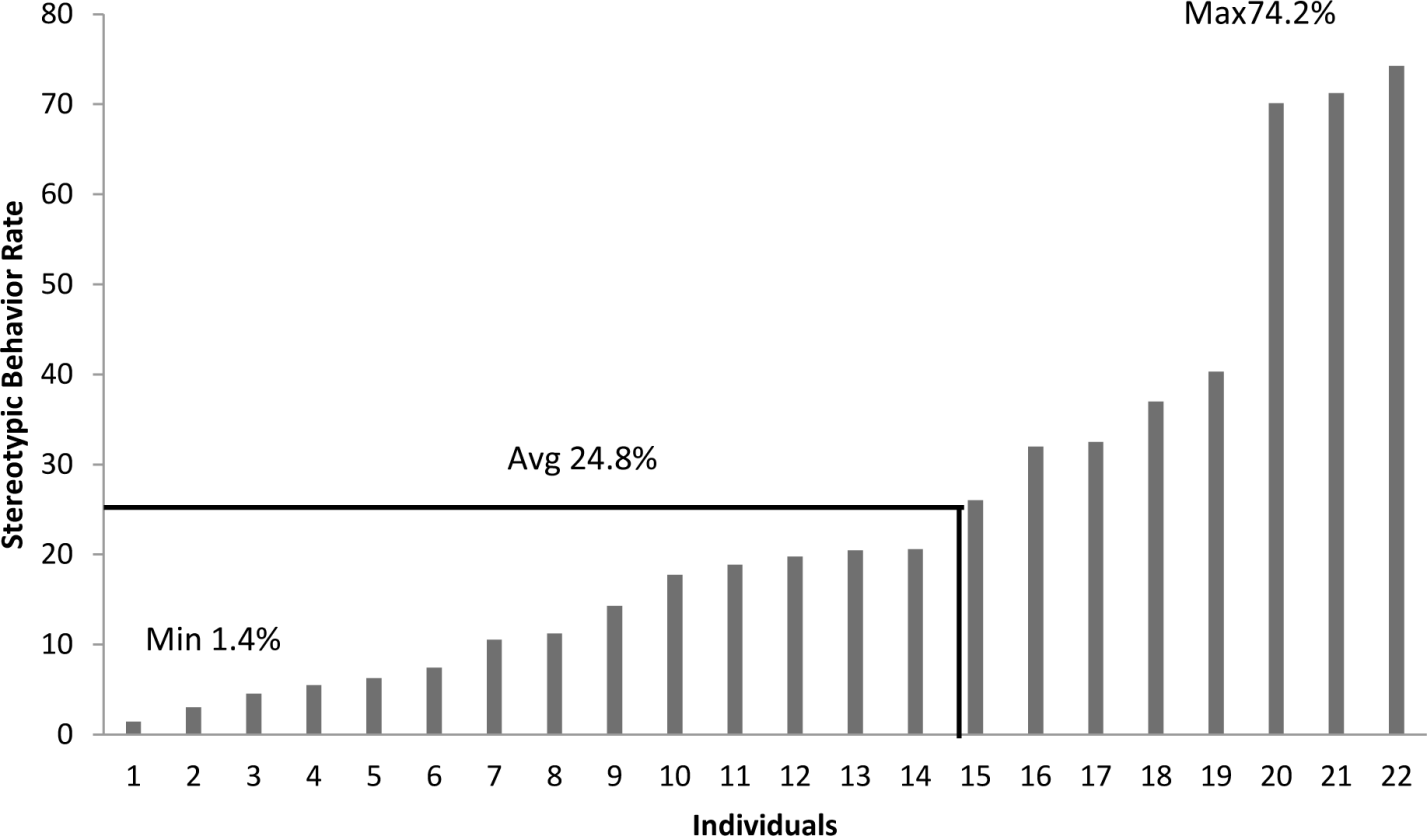
Nighttime stereotypic behavior performance rates calculated as a percentage of active time for individual elephants. The average stereotypic behavior rate in the full population was 24.8%, the min 1.4%, and the max 74.2%.

During the daytime 85.4% (N = 76) of the elephants performed stereotypic behavior, with rates ranging between 0.5% and 68.1% of active time (Fig 5). Of these, 35 were African (N = 4 males, N = 31 females) and 41 were Asian (N = 8 males, N = 33 females). On average, stereotypic behavior was performed at the following rates (proportion of active behavior observations ± SE) during the daytime: Africans (8.5% ± 2.3), Asians (21.4% ± 2.9), males (28.1% ± 5.2), and females (12.9% ± 2.1). The majority of daytime stereotypic behaviors (91.0%) were motor movements (as described in Table 2). During the nighttime 68.8% (N = 22) of the elephants performed stereotypic behavior, with rates ranging between 1.43% and 74.2% (Fig 6). Of the elephants that performed stereotypic behavior, 9 were African (N = 1 male, N = 8 females) and 13 were Asian (N = 3 males, N = 10 females). On average, stereotypic behavior was performed at the following rates (proportion of active behavior observations ± SE) during the nighttime: Africans (9.9% ± 3.1), Asians (35% ± 4.8), males (35.8% ± 1.3), and females (22.3% ± 5).

### Stereotypic Behavior Models

Stereotypic behavior rate risk ratios were the primary statistics produced by each of our epidemiological analyses. Even though risk ratios describe the effect of an independent variable on the dependent variable, it is important to note that this effect is conditional on the effects of the other independent variables in each model. To facilitate interpretation of the risk ratios associated with our results, we have included several figures which illustrate the effect of specific input variables when all other input variable values are held constant at their average values.

**Fig 7:**
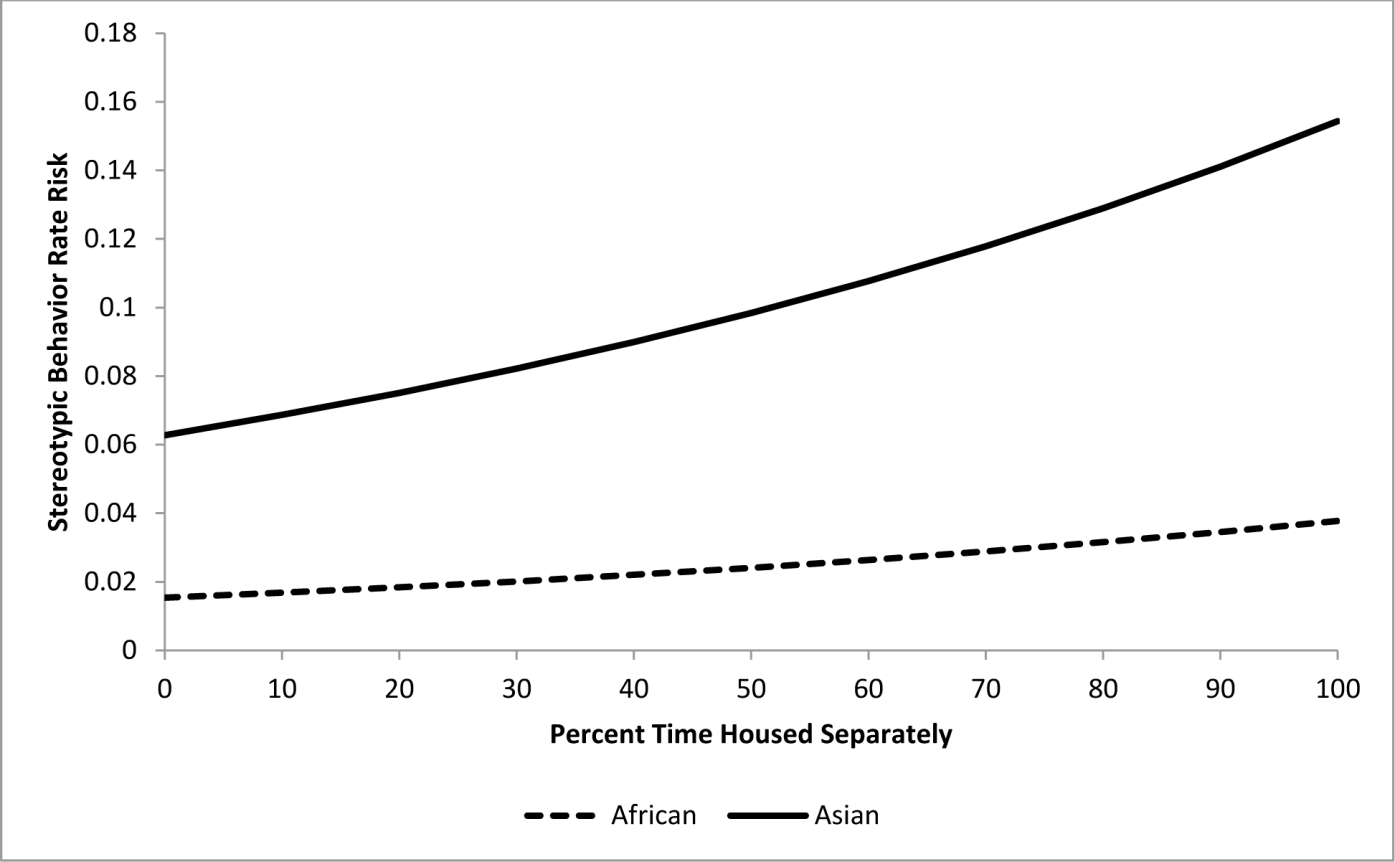
Risk increase for stereotypic behavior rate by Percent Time Housed Separately for African and Asian elephants: Percent Time with Juveniles, Percent Time Managed, and Transfers are all held constant at the average levels (13.0%, 54.1%, and 3.3 respectively). Values on the X-axis reflect the range of Percent Time Housed Separately scores seen within our sample population.

**Fig 8:**
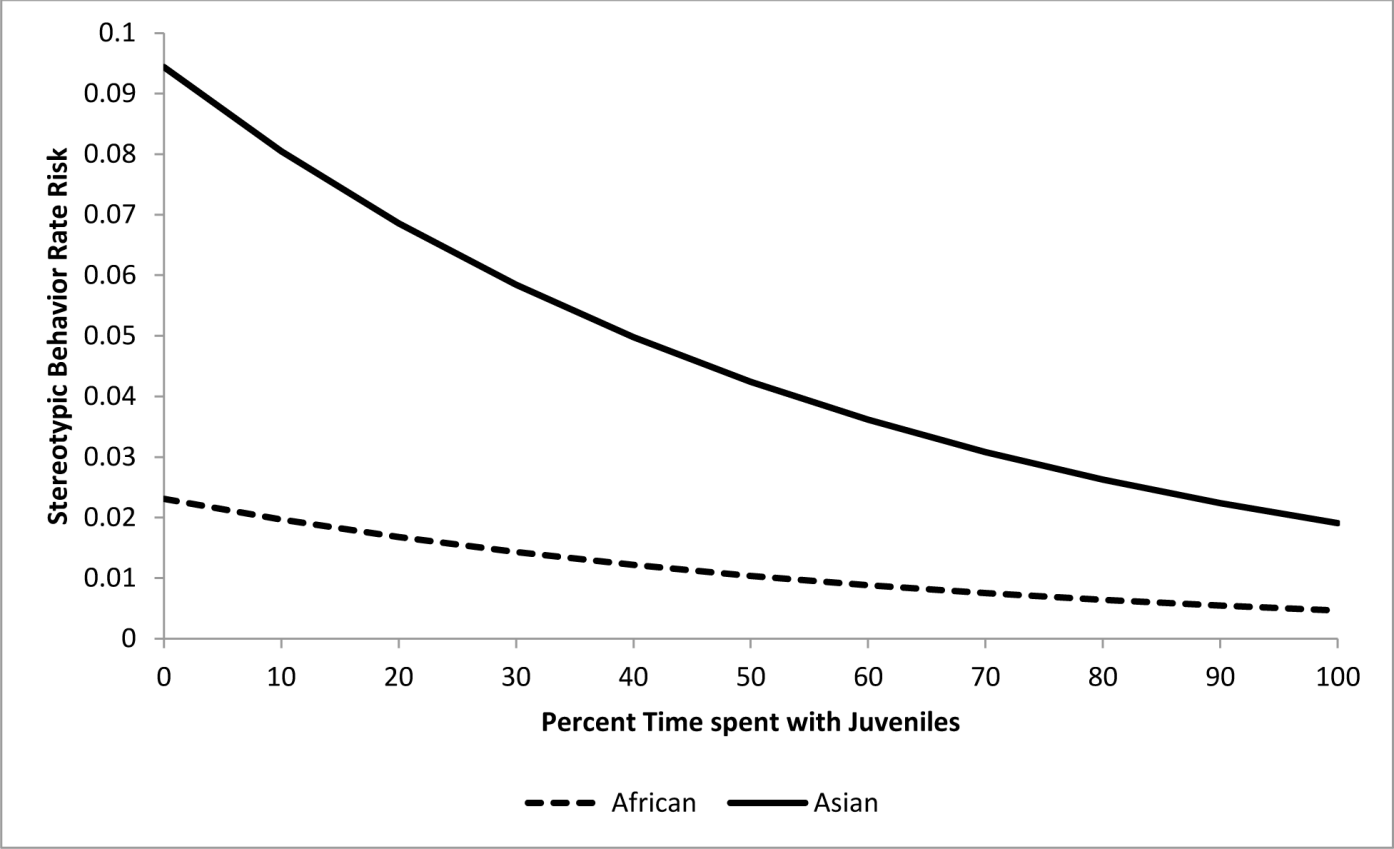
Risk increase for stereotypic behavior rate by Percent Time with Juveniles for African and Asian elephants: Percent Time Housed Separately, Percent Time Managed, and Transfers are all held constant at the average levels (22.3%, 54.1%, and 3.3 respectively). Values on the X-axis reflect the range of Percent Time with Juveniles scores seen within our sample population.

**Fig 9:**
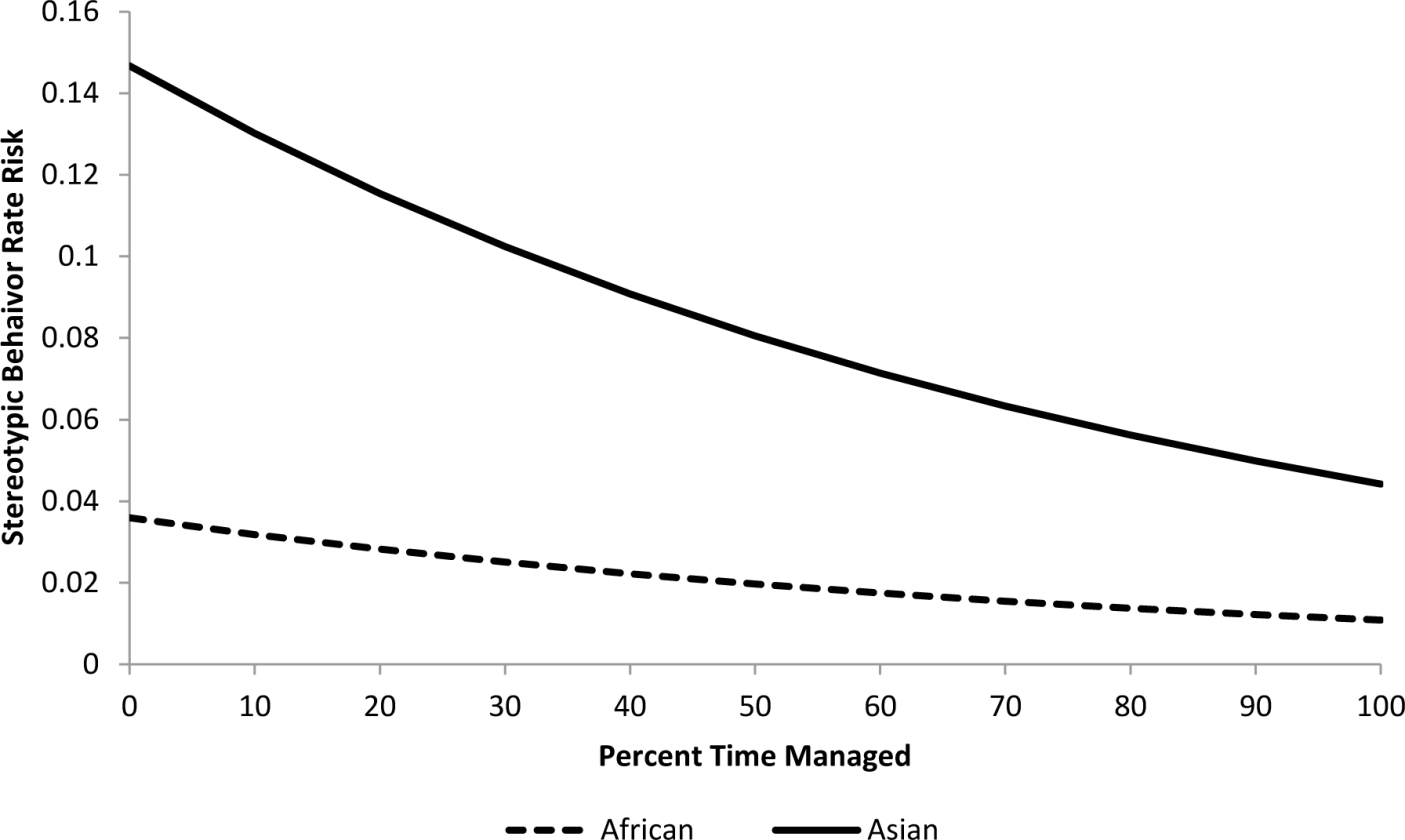
Risk increase for stereotypic behavior rate by Percent Time Managed for African and Asian elephants: Percent Time Housed Separately, Percent Time with Juveniles, and Transfers are all held constant at the average levels (22.3%, 13.0%, and 3.3 respectively). Values on the X-axis reflect the range of Percent Time Managed scores seen within our sample population.

**Fig 10:**
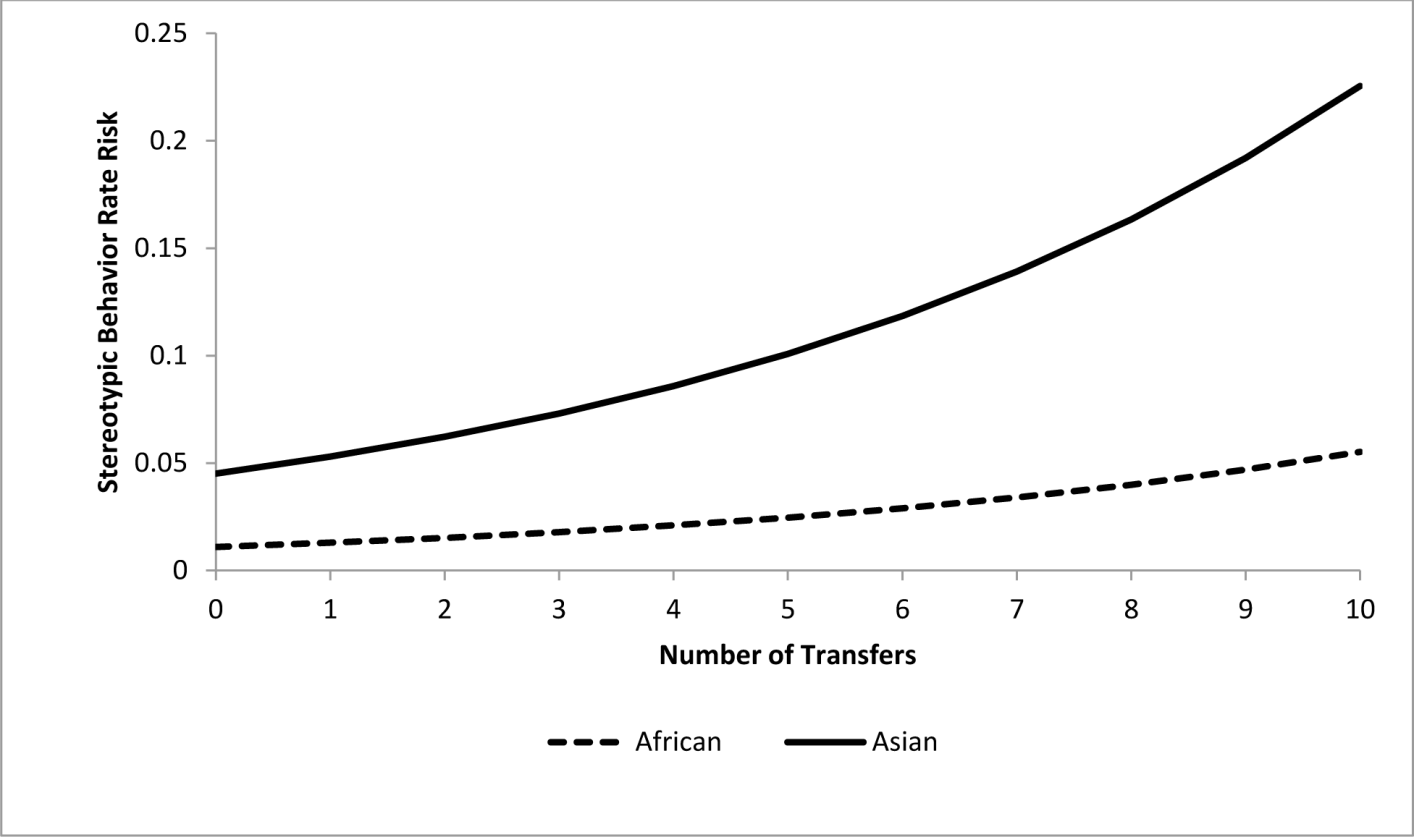
Risk increase for stereotypic behavior rate by Transfers for African and Asian elephants: Percent Time Housed Separately, Percent Time with Juveniles, and Percent Time Managed, and Transfers are all held constant at the average levels (22.3%, 13.0%, and 54.1% respectively). Values on the X-axis reflect the range of Transfers scores seen within our sample population.

For the epidemiological analysis of daytime stereotypic behavior rates, 21 of 41 tested variables were associated (*p*<0.15) with stereotypic behavior rates at the univariate level (Table 3). In the final model, a combination of five variables significantly (*p<*0.05) contributed to stereotypic behavior rate risk (Tables 4 and 5). Asian elephants were at 4.1 times greater risk than African elephants. The risk of performing stereotypic behavior at or above an individual’s observed rate was increased by 9% for every 10% increase in time housed separately (Percent Time Housed Separately) (Fig 7), but was reduced by 15.4% for every 10% increase in time spent with juvenile elephants (Percent Time with Juveniles) (Fig 8). Additionally, an increase of 10% in time spent engaged in managed activities (Percent Time Managed) reduced risk by 11.5% (Fig 9). Having experienced transfers between institutions (Transfers) corresponded with a 17.5% increase in risk for every additional transfer experienced (Fig 10). Age contributed to the model as a non-significant confounder of Percent Time with Juveniles, Percent Time Managed, and Transfers. Sex also contributed to the models as a non-significant confounder of Percent Time Housed Separately, Percent Time Managed, and Transfers.

**Table 3:** Independent variables tested for association with daytime stereotypic behavior rates and statistics associated with the univariate negative binomial regression models.

Variable Class	Variable	Reference	N^1^	β-coefficient	Relative Risk	p*
	Animal Contact	None	171	-0.263	0.768	<0.001*
	Herd Size	None	180	-0.041	0.959	0.553
	Percent Time Housed Separately	None	177	0.012	1.012	<0.001*
	Percent Time Mixed Age Group	None	177	-0.010	1.012	<0.001*
Social^2^	Percent Time Single Age Group	None	177	-0.006	1.012	0.065*
	Percent Time Single Sex Group	None	177	-0.007	1.012	0.016*
	Percent Time w/ Juveniles	None	177	-0.018	0.982	<0.001*
	Relative Social Experience Change	None	175	-0.781	0.592	0.240
	Social Group Contact	None	177	-0.162	0.850	0.130^
	Social Experience	None	172	-0.568	0.567	<0.001*
	Environment Contact	None	177	0.048	1.049	0.324
	Percent Indoor	None	177	-0.005	0.995	0.082
	Percent In/Out Choice	None	169	-0.012	1.004	0.158**
	Percent Outdoor	None	178	<0.01	1.003	0.917
	Percent Time on Hard Substrate	None	177	-0.002	1.003	0.832
Housing^2^	Percent Time on Dirt Substrate	None	177	0.001	1.003	0.858
	Percent Time on Soft Substrate	None	177	0.003	1.003	0.598
	Percent Time on Soft Substrate or Dirt	None	177	0.002	1.003	0.806
	Space Experience	None	169	-0.010	0.990	<0.001*
	Space Experience Per Elephant	None	165	0.004	1.004	0.447
	Space Experience Indoor	None	177	-0.048	0.953	0.292
	Space Experience Outdoor	None	175	-0.006	0.994	0.017*
	Enrichment Diversity	None	162	1.351	3.862	0.105^
	Enrichment Program	None	166	0.129	1.138	0.259
		Ref = (less than hour per week	30	0	1.000	
	Exercise Per Week	one to three hours per week	73	0.832	2.298	0.184
		7 to 10 hours per week	36	0.552	1.736	0.429
		Greater than 14 hours per week	10	1.068	2.909	0.187
	Feed Day	None	162	-0.105	0.900	0.020*
		Ref = Predictable	24	0		
Management^3^	Feeding Predictability	Semi-predictable	112	0.136	1.146	0.757
		Unpredictable	30	-0.705	0.494	0.212
	Alternate Feeding Methods	None	176	0.892	2.440	0.267
		0%	63	0		
	Percent Guide Interaction Time (0% of interactions involve a guide	1%-30%	48	-0.187	0.829	0.567
		31%-80%	11	-0.979	0.376	0.025*
		>80%	38	-0.423	0.655	0.354
	Percent Time Managed	None	160	0.010	1.010	0.079^
		Ref = Sometimes/ frequent	28	0		
	Rewarding Stimuli Techniques Score (Sometimes/frequent experience)	Frequent	63	0.839	2.315	<0.001*
		Frequent/ very frequent	56	1.040	2.829	<0.001*
		Ref = less than one hour per week	72	0		
	Walk Week: (less than one hour per week)	one to three hours per week	59	0.059	1.061	0.863
		five to seven hours per week	15	-0.261	0.771	0.602
	Experience Birth	None	172	0.061	1.063	0.175
	Experience Death	None	170	0.094	1.099	<0.001*
Life History^4^	Separation Age	None	18	0.041	1.041	0.259
	Separation by Age	None	18	-1.101	0.333	0.322
	Transfers	None	176	0.156	1.168	<0.001*
	Age	None	180	0.043	1.044	<0.001*
	Origin	Ref = Imported from home-range	152	0		
		Captive born	28	-0.089	0.915	0.841
Demographic^4^	Sex	Ref = Male	31	0		
		Female	149	-0.660	0.517	0.017*
	Species:	African	96	0		
		Asian	84	1.209	3.349	<0.001*
Other	Season: (winter)	None	180	0.116	1.123	0.259

*Variable was retained for use in the model building process when *p*<0.05.

^Variable was retained for use in the model building process when *p*<0.15.

**Variable predicted stereotypic behavior rate (*p*<0.15) after bivariate testing with confounding demographic variables, and was retained for use in the model building process.

^1^ N is the number of observations used in the repeated measures analysis

^2^ Detailed descriptions of Social and Housing variables can be found in Meehan et al. [17].

^3^ Detailed descriptions of Management variables can be found in Greco et al. [16].

^4^ Detailed descriptions of Life History and Demographic variables can be found in Prado-Oviedo et al. [20].

**Table 4:** Daytime model for variables associated with stereotypic behavior rate risk (N = 154, QIC = -8481).

Variable	β-coefficient	Standard Error	95% Confidence Limits		p*	Relative Risk
**Intercept**	-3.851	0.530	-4.890	-2.811	< .001*	
**Percent Time Housed Separately**	0.009	0.003	0.003	0.015	< .001*	1.009
**Percent Time w/ Juveniles**	-0.016	0.004	-0.023	-0.008	< .001*	0.985
**Percent Time Managed**	-0.012	0.005	-0.021	-0.002	0.018*	0.988
**Transfers**	0.161	0.056	0.051	0.272	< .001*	1.175
**Age**	0.019	0.014	-0.009	0.047	0.190	1.019
**Sex (Male)**	0					
**Sex (Female)**	0.134	0.306	-0.465	0.733	0.661	1.143
**Species (African)**	0					
**Species (Asian)**	1.408	0.287	0.846	1.970	< .001*	4.087

*Significant when p<0.05

**Table 5:** Descriptive statistics for variables in the daytime model.

Variable	N^1^	Mean	Std Dev	Min	Median	Max
**Percent Time Housed Separately**	177	22.3	37.9	0	0	100
**Percent Time w/ Juveniles**	177	13.0	30.4	0	0	100
**Percent Time Managed**	160	54.1	23.1	0	50	99
**Transfers**	176	3.3	2	0	3	10
**Age**	180	36.3	9.6	61	35.5	16
**Sex**	Males:31, Females: 149					
**Species**	Africans: 96,Asians: 84					

^1^N is the number of observations used in the repeated measures analysis

**Fig 11:**
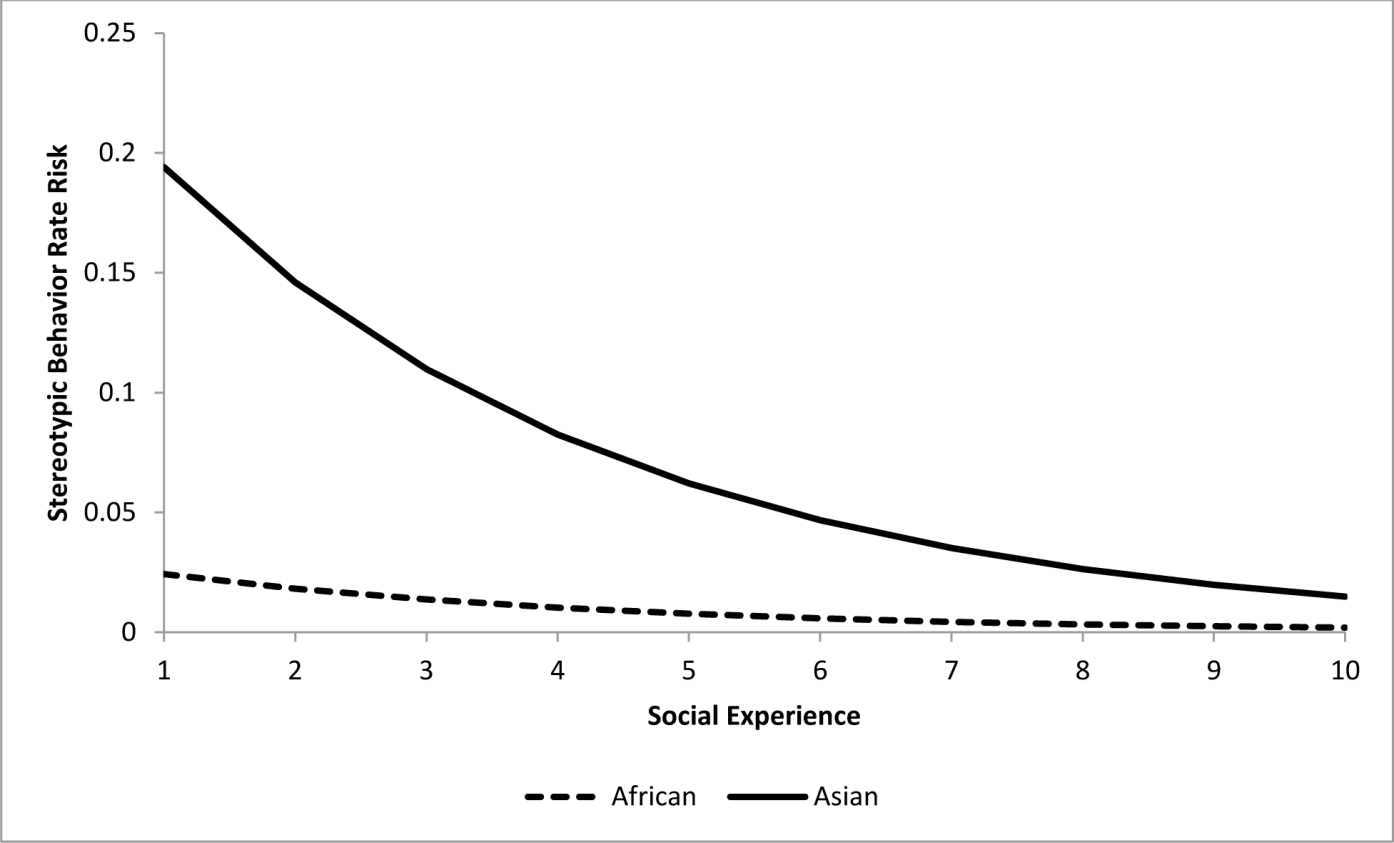
Risk increase for stereotypic behavior rate by Social Experience for African and Asian elephants: Percent Time with In/Out Choice, and Transfers are both held constant at the average levels (31.7% and 3.2 respectively). Values on the X-axis reflect the range of Social Experience scores seen within our sample population.

**Fig 12:**
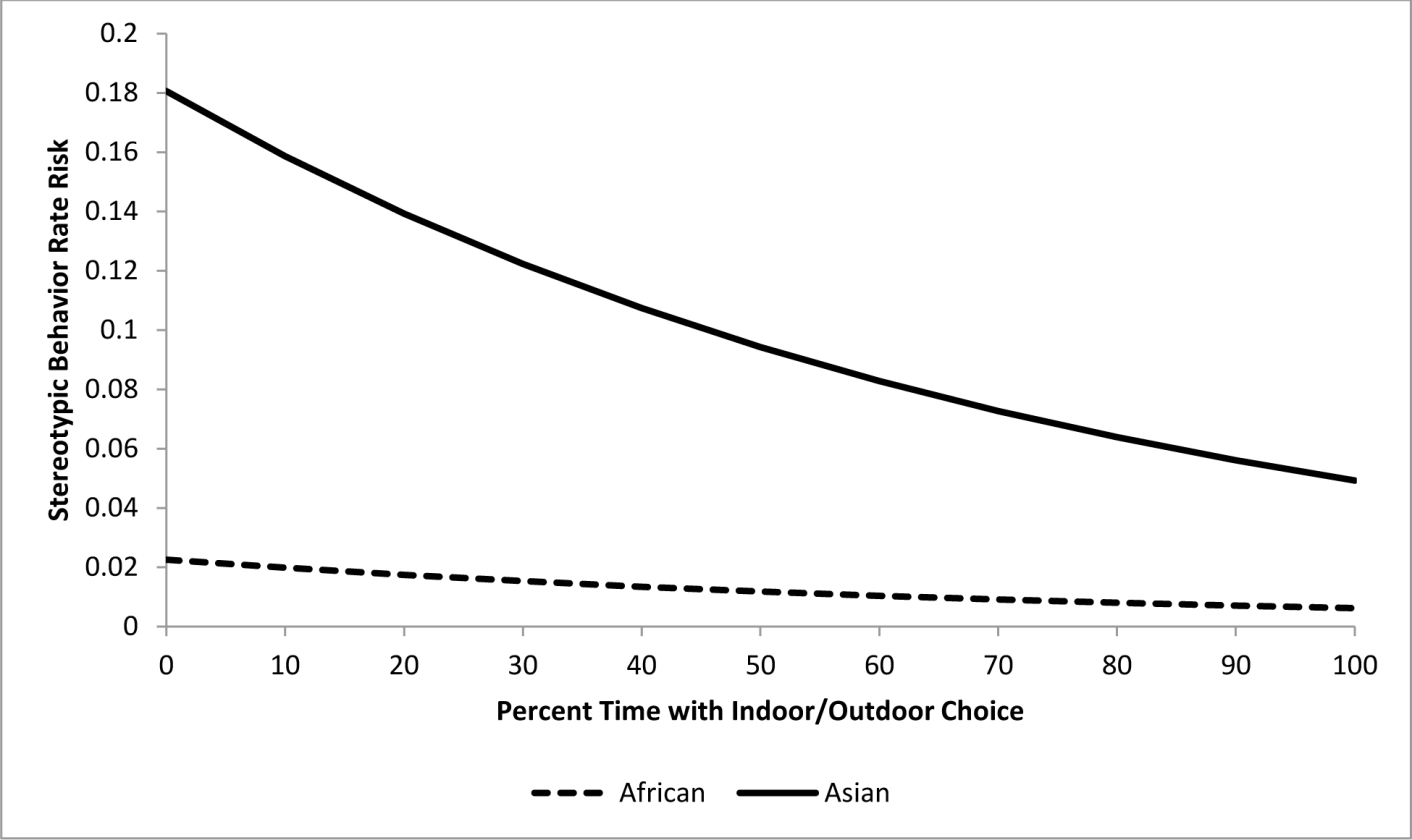
Risk increase for stereotypic behavior rate by Percent Time In/Out Choice for African and Asian elephants: Social Experience and Transfers are all held constant at the average levels (2.7 and 3.2 respectively). Values on the X-axis reflect the range of Percent Time In/Out Choice scores seen within our sample population.

**Fig 13:**
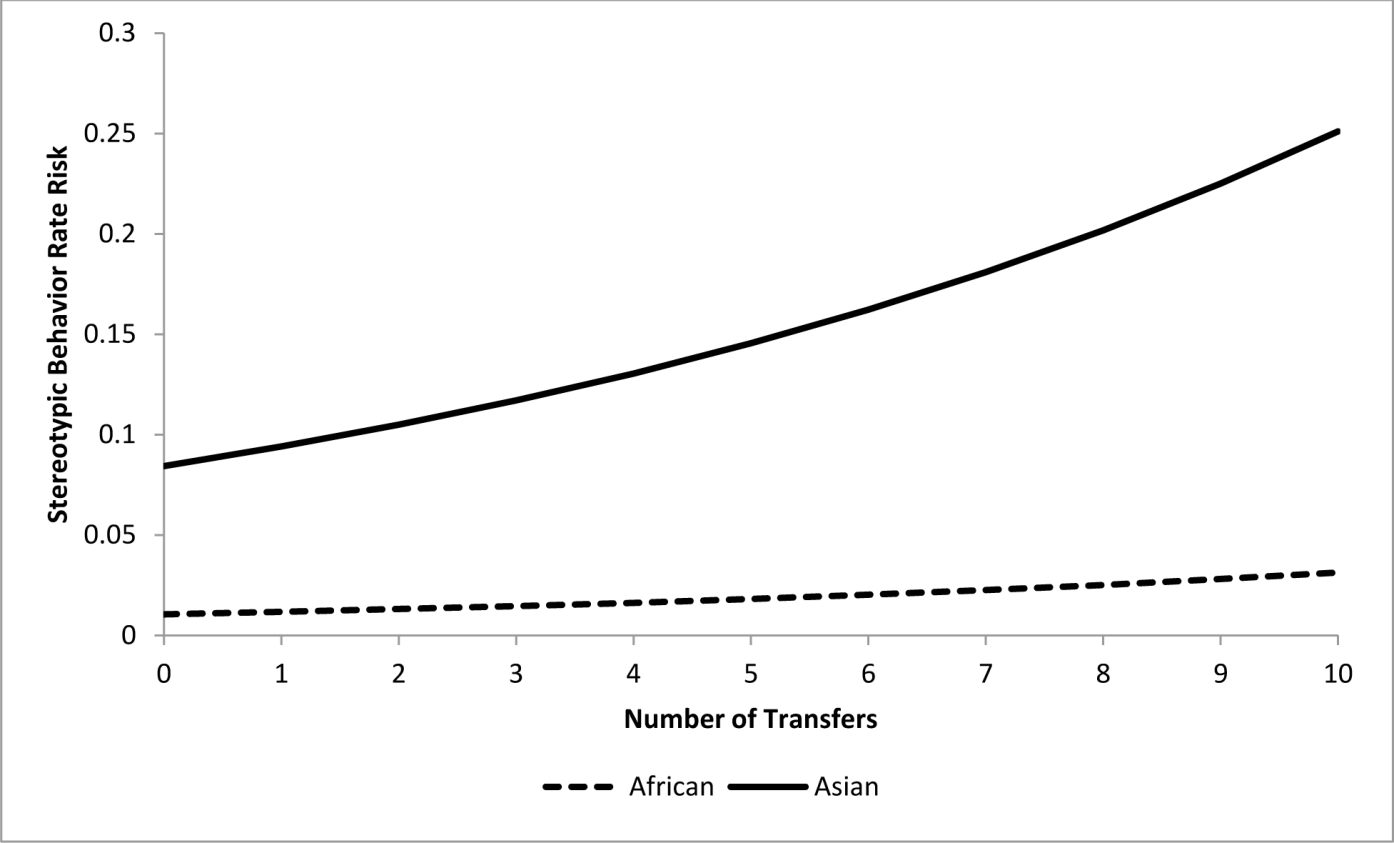
Risk increase for stereotypic behavior rate by Transfers for African and Asian elephants: Social Experience and Percent Time In/Out Choice are all held constant at the average levels (2.7 and 31.7% respectively). Values on the X-axis reflect the range of Transfers scores seen within our sample population.

For the epidemiological analysis of nighttime stereotypic behavior, 15 of 22 tested variables were associated (*p<*0.15) with stereotypic behavior rates at the univariate level (Table 6). In the final model, a combination of four variables significantly (*p<*0.05) contributed to stereotypic behavior rate risk (Tables 7 and 8). Asian elephants were at eight times greater risk than African elephants. A one unit increase in Social Experience score during the nighttime corresponded with a 24.8% reduction in risk (Fig 11). Risk decreased by 12.7% for every 10% increase in time spent during the nighttime in environments with an indoor/outdoor choice (‘Percent Time In/Out Choice’) (Fig 12). Transfers again corresponded with an increased risk, with an 11.5% increase for every additional transfer experienced (Fig 13). Finally, Age contributed to the model as a non-significant confounder of Percent Time In/Out Choice and Transfers.

**Table 6:** Independent variables tested for association with nighttime stereotypic behavior rates and statistics associated with the univariate negative binomial regression models.

Variable Class	Variable	Reference	N	β-coefficient	Relative Risk	p*
	Animal Contact	None	32	-0.399	0.671	<0.001*
	Herd Size	None	32	-0.077	0.926	0.280
Social^1^	Percent Time Housed Separately	None	32	0.012	1.012	<0.001*
	Relative Social Experience Change	None	32	0.901	2.461	0.248
	Social Group Contact	None	32	-0.956	0.384	<0.001*
	Social Experience	None	32	-0.636	0.529	<0.001*
	Percent Indoor	None	32	0.016	1.016	<0.001*
	Percent In/Out Choice	None	32	0.002	1.002	0.746**
	Percent Outdoor	None	32	-0.012	0.988	0.033
	Percent Time on Hard Substrate	None	32	0.010	1.010	0.224
Housing^1^	Relative Space Experience Change	None	32	0.721	2.055	0.195**
	Space Experience	None	32	-0.146	0.864	0.012*
	Space Experience Indoor	None	32	0.134	1.144	0.161**
	Space Experience Outdoor	None	32	-0.010	0.990	0.001*
	Enrichment Diversity	None	32	0.022	1.022	0.991
Management^2^	Enrichment Program	None	32	0.387	1.473	0.269
	Feed Night	None	32	-0.403	0.668	0.027
	Alternate Feeding Types	None	32	4.039	56.741	0.001*
Life History ^3^	Transfers	None	32	0.071	1.074	0.272**
	Age	None	32	0.045	1.046	0.136^
	Origin:	Imported from home-range	29	0		
		Captive born	3	-0.256	0.774	0.472
Demographic ^3^	Sex:	Male	6	0		
		Female	26	-0.430	0.651	0.286
	Species:	African	19	0		
		Asian	13	2.018	7.520	<0.001*

*Variable was retained for use in the model building process when *p*<0.05.

^Variable was retained for use in the model building process when *p*<0.15.

**Variable predicted stereotypic behavior rate (*p*<0.15) after bivariate testing with confounding demographic variables, and was retained for use in the model building process.

^1^ Detailed descriptions of Social and Housing variables can be found in Meehan et al. [17].

^2^ Detailed descriptions of Management variables can be found in Greco et al. [16].

^3^ Detailed descriptions of Life History and Demographic variables can be found in Prado-Oviedo et al. [20].

**Table 7:** Nighttime model describing variables associated with stereotypic behavior rate risk (N = 32, QIC = -2358).

Variable	β-coefficient	Standard Error	95% Confidence Limits		p*	Relative Risk
**Intercept**	-3.372	0.703	-4.749	-1.995	< .001*	
**Social Experience**	-0.285	0.138	-0.555	-0.015	0.039*	0.752
**Percent Time In/Out Choice**	-0.013	0.005	-0.023	-0.003	0.013*	0.987
**Transfers**	0.109	0.051	0.009	0.209	0.033*	1.115
**Age**	0.023	0.020	-0.015	0.062	0.229	1.024
**Species (African)**	0					
**Species (Asian)**	2.081	0.436	1.226	2.936	< .001*	8.015

*Significant when p<0.05

**Table 8:** Descriptive statistics for variables in the nighttime model.

Variable	N	Mean	Std Dev	Min	Median	Max
**Social Experience**	32	2.7	2.4	1	2	11
**Percent Time In/Out Choice**	32	31.7	37.7	0	15	100
**Transfers**	32	3.2	2	0	3	10
**Age**	32	35.3	9.5	18	34	58
**Species**	Africans: 19, Asians: 13					

### Seasonal and Time of Day Effects

Predictive analyses demonstrated that stereotypic behavior rates were consistent across season and housing period (daytime vs nighttime). Thus, winter daytime rates significantly predicted summer-daytime rates (β = 5.62, *p*<0.01), winter daytime rates significantly predicted fall nighttime rates (β = 4.66, *p*<0.01), and summer daytime rates significantly predicted fall nighttime rates (β = 2.64, *p*<0.01).

### Comparison of Significant Independent Variables between Sub-population and full-population

For all assessed independent variables that contributed to the final models, our population did not significantly differ from the larger adult elephant population (older than 12 years of age) and as such are a representative sample of North American zoo elephants (Table 9).

**Table 9:** Comparison of significant independent variables between the sub-population in our study (N = 89 daytime, N = 32 nighttime) and the adult North American zoo elephant population (N = 217).

**Variables included in the daytime model**		
	Full	Day Behavior
Percent Time Housed Separately^1^	28.0	30.6
Percent Time Managed^1^	52.6	54.1
Percent Time with Juveniles^1^	12.8	13.4
Transfers^1^	3.1	3.4
Age^1^	35.0	36.3
Male^2^	32	15
Female^2^	185	74
Asian^2^	104	47
African^2^	113	42
**Variables included in the nighttime model **		
	Full	Night Behavior
Percent Time In/Out Choice^1^	16.6	18.5
Social Experience^1^	2.4	2.7
Transfers^1^	3.1	3.2
Age^1^	35.0	35.3
Asian^2^	104	13
African^2^	113	19

^1^Least squared means statistic

^2^Chi Square statistic. No subgroup comparisons were significantly different from the full population at *p*<0.05

## Discussion

Our study provides the first large scale assessment of behavioral time budgets and epidemiological analyses of the risk factors associated with the stereotypic behavior rates of zoo elephants. In addition to being the most comprehensive project of its kind, the elephants in our subsample were shown to be representative of the larger population of adult elephants housed in accredited North American zoos. Thus, the results and principles derived from this study should be broadly applicable. In the discussion that follows, we explore the range of social, housing, management, and life history risk factors highlighted by our models and consider each of these variables in the context of enhancing zoo elephant care and welfare.

### Behavioral Time Budgets

The behavioral time budgets confirmed previous findings [6,9,11,12] that stereotypic behavior, along with feeding and rest, are the three behaviors most commonly performed by zoo elephants. We also confirmed that zoo elephants shift towards less active behavior during the nighttime, spending 60% of their nighttime period active as opposed to the 80% spent active during the daytime. Lower locomotion and self-maintenance behavior rates contributed to reductions in activity during the nighttime. These findings are consistent with descriptions of the circadian cycles of elephants in both managed care [9] and in the wild [48].

### Stereotypic Behavior

Despite reduced activity levels at night, we found no differences in stereotypic behavior rates between daytime and nighttime. We similarly found no differences in stereotypic behavior rates between seasons (winter, summer, or fall). Such consistency in rates might be attributable to the neurophysiological changes that often underlie the performance of stereotypic behavior [5]. These changes are semi-permanent in nature, reduce behavioral flexibility, and can require prolonged interventions to change. [5]. Thus, once an animal develops stereotypic behaviors, it may not be possible to eradicate the behavior completely, but may be possible to change performance rate [5]. In the text that follows, we discuss a number of variables that our epidemiological analyses identified as being important predictors of stereotypic behavior. Understanding how these variables relate to stereotypic behavior performance may help zoo professionals reduce the rates at which their elephants perform stereotypic behavior and reduce the likelihood of stereotypic behavior development in elephants that do not perform these behaviors.

### Social Variables

Our final stereotypic behavior models contained three social variables, two of which (Social Experience and Percent Time with Juveniles) reduced stereotypic behavior rate risk, while the third, Percent Time Housed Separately, increased risk. Only this last variable can be directly compared to results from other studies. Spending time housed separately from social partners has been identified as a risk factor for stereotypic behavior in a wide variety of animals (as reviewed in Mason and Rushen [5]), including elephants [15], and likely contributes to stereotypic behavior rate because separation frustrates an elephant’s motivations to engage in social behavior [5]. Unfortunately, too few of the elephants in our population contributed data describing the amount of time they spent separated via porous barriers through which they could see and/or touch one another (Percent Time Housed Separately with Restricted Physical Access [17], a practice considered to be socially stimulating [49]). Thus, building stable models with this variable was not possible, making it impossible for us to determine whether restricted contact and full social separation pose different risks.

In contrast, directly sharing space with suitable partners often protects against stereotypic behavior development in social species (as reviewed in Mason and Rushen [5]). Thus, it is perhaps unsurprising that that Social Experience scores (spending more time with larger numbers of herdmates) corresponded with reduced stereotypic behavior rate risk. The protective effect associated with this variable may reflect the fact that large groups increase environmental variability and complexity, increasing cognitive flexibility and stimulating more opportunities for behavioral expression [50]. Larger groups may also facilitate social buffering of stress by providing captive animals with more opportunities to manage social challenges and/or to choose among more social partners [51,52]**.** Perhaps more surprising was the protective effect of Percent Time with Juveniles, revealing that the age of social partners is important. Spending time with juveniles might stimulate play or teaching behavior in older herdmates [53]. Fully understanding exactly how young conspecifics reduce stereotypic behavior rate risk in adults will require more targeted research.

Although the daytime and nighttime models did not contain the same social variables, we believe that all three variables are broadly applicable predictors of stereotypic behavior rates. This is especially apparent with Social Experience and Percent Time Housed Separately, which predicted stereotypic behavior rate risk throughout the model building process. Percent Time with Juveniles could not be reliably tested in the nighttime model, since only 6 of the 32 elephants in our night sample spent time with young at night. However, based on its effect on stereotypic behavior rate risk in the daytime model, access to juveniles would likely reduce risk at night as well.

### Housing Variables

The only housing variable that was present in a final model was Percent In/Out Choice, which was associated with reduced risk. This variable describes the percent of time an elephant is housed in environments where there are both indoor and outdoor options it can choose between. Although this variable’s protective effect was most noticeable in the final nighttime model, it also made it into the final round of the model building process with the daytime, implying that its effect is more broadly applicable. We hypothesize that the relationship between Percent In/Out Choice and reduced stereotypic behavior rate risk may be attributable to the increased complexity or quality inherent to “mixed” (indoor/outdoor choice) environments, as such environments provide access to a greater diversity of features and resources (e.g., cooler/warmer microclimates, social/environmental refuge, etc.) [54], and also afford elephants the opportunity to choose between environmental options. Whether it is actually this element of control that is key to reducing stereotypic behavior rate risk is difficult to determine, because only a few non-elephant studies investigating mixed environments and stereotypic behavior have ever been conducted [54–56]. Two of the three studies [54,56] found that providing free access to indoor enclosures resulted in the study animals (panda and polar bears, respectively) preforming lower stereotypic behavior rates. Both authoring groups attribute this reduction to the availability to make choices [54,56]. However, the indoor environments to which the bears were given access may have provided additional complexity (e.g., cooler microclimates, social refuge), demonstrating the difficulty associated with teasing apart the potential contributing factors present in studies conducted in active zoo environments.

The inclusion of Percent In/Out Choice in our final nighttime mode is in line with our hypotheses. However, prior to conducting our analyses we expected that some of the Space Experience variables–which weighted the size of various environments by the amount of time elephants spent in those types of environments to account for dynamic housing practices where elephants are shifted between areas throughout the day and night–would be among the most important housing variables in both the daytime and nighttime models. This expectation was based on findings from previous studies showing that stereotypic behavior in elephants is closely linked to the amount of space to which they have access [3,13,14]. Two Space Experience variables (Overall and Outdoor) consistently predicted reduced stereotypic behavior rates throughout the model building process, lending some support to previous elephant welfare studies linking the size of enclosures to stereotypic behavior, but neither of these variables contributed to our strongest model. While amount of space per se was not included in our models, it is important to note that there needs to be sufficient space to support social group management, especially large multi-generational herds. Thus, despite the fact that no space experience variables were included in our final models, it would be inadvisable to overlook these types of variables in future elephant welfare studies.

### Management Variables

Percent Time Managed was the only management variable in the final daytime stereotypic behavior model. The management data [16] showed that the elephants spent an average of just over 50% of their daytime hours in managed activities including exercise sessions, foot and skin care, and training routines. Although their goals vary, these activities are all alike in that the elephants are under behavioral control of a human caretaker during their performance [16]. In our model, as elephants spent more time in this managed condition, their stereotypic behavior rate risk decreased. Since our data collection protocol required that the behavior observations occur outside of managed time, this association is not simply the result of elephants having less time available to perform stereotypy. Rather, the protective influence of Percent Time Managed may be attributable to the social relationships that the elephants develop with their caretakers. A number of studies of livestock and zoo animals have shown that positive and predictable relationships with care staff are associated with reduced stress and improved general welfare [57,58]. Additionally, when human-animal relationships involve positive reinforcement training, as did most relationships experienced by the elephants in our population [16], the quality of the relationship is further increased [26,59].

Despite the fact that enrichment is commonly linked to reduced stereotypic behavior [23], neither the Enrichment Program variable nor the Enrichment Diversity variable predicted stereotypic behavior rates very well. Enrichment Program was eliminated at the univariate level, showing no relationship to stereotypic behavior rate. Enrichment Diversity was included in a number of early models, but quickly fell out as the model building process proceeded. The weak predictive role of these variables could be attributable to the fact that both of these enrichment variables, unlike the other significant variables in our models, are program-level variables which characterize the practices of care staff rather than approximating the experiences of individual elephants. In addition to linking enrichment variables to individual experience, it may be beneficial for future studies to develop enrichment variables that describe the elephants’ use of enrichment, rather than enrichment item presentation. Since many of the variables in our models demonstrate the importance of the social environment on stereotypic behavior performance, it seems possible that zoo enrichment activities that are used to enhance elephants’ social opportunities could have particularly strong impacts on stereotypic behavior rates.

### Life History Variables

Our final stereotypic behavior models contained one life history variable, Transfers (the number of inter-zoo transfers an elephant has experienced), which corresponded with increased stereotypic behavior rate risk. Considering the amount of research suggesting that life history events play major roles in the development and performance of stereotypic behavior (as reviewed in Mason and Rushen [5]) inclusion of the Transfers variable in both final models is unsurprising. Stressors associated with transport conditions (e.g., social separation, spatial restriction, uncontrollable noise/environmental exposure, food restriction), and those that follow transport (e.g., exposure to new environments and integration with new social partners), have been linked to stereotypic behavior performance in other species and in other contexts [5]. Furthermore, stereotypic behaviors are often performed before, during, and/or after transport events by animals in other taxa (tigers: [60]; horses: [61]). The influential effect of social separation on the performance of stereotypic behavior by elephants has also been shown in our analyses (see discussion regarding Percent Time Housed Separately). Two case studies [62,63] also have shown that transfers can have a transient effect on zoo elephants’ acute physiological indicators of stress, and an analysis of Asian elephants in European zoos suggests that the stress associated with transfers may have contributed to the association that was found in that study between transfers and increased mortality risk [64].

Early maternal separation is widely reported to be a risk factor for stereotypic behavior in a number of species [28], including elephants [15]. Thus, we hypothesized that Separation Age (the age at which elephants are separated from their mothers by death or transfer) would correspond with increased stereotypic behavior risk rate in our models. Unfortunately, Separation Age data were only available for 10 elephants in our study population. It was impossible to construct stable models with such a small sample size. However, we believe that the timing of mother-offspring separation should continue to be investigated as a potential predictor of elephant stereotypy.

### Demographic Variables

Asian elephants were at a greater risk of performing stereotypies at higher rates than African elephants. It has been suggested by elephant naturalists and care professionals that Asian elephants may be more susceptible to developing stereotypic behavior because many spent their early developmental years in potentially stressful environments, like circuses and/or Southeast Asian working camps [4]. This hypothesis seems particularly plausible considering the extensive research demonstrating the lasting effects of early rearing conditions on stereotypic behavior (as reviewed in Mason and Rushen [5]) and could be tested more explicitly in longitudinal studies. Additionally, genetic differences have been shown to influence how susceptible animals are to developing stereotypies in other taxa [5,33,65]. The Asian and African species diverged from a common ancestor between 4.2 and 9 million years ago [66] and as such, there could be evolutionary biology/genetic factors predisposing Asian elephants to stereotypic behavior.

## Conclusions

Stereotypic behavior is often used as an important indicator of compromised welfare. Although there have been a number of studies examining stereotypic behavior in captive elephants, our study is the first to use epidemiological methodologies with a robust sample size representative of the North American zoo population. Like several previous studies [11,15,22], our final models point toward the social environment as having the most important impact on stereotypic behavior rates. Three of the seven unique variables included in our final models were social variables, with two (Percent Time with Juveniles and Social Experience) contributing to reduced stereotypic behavior rate risk, and the other (Percent Time Housed Separately) contributing to increased risk. In addition, it is our interpretation that the associations of Percent Time Managed and Transfers with stereotypic behavior rate risk are likely to be related to sociality as well. For example, the regular human-animal social interactions described by Percent Time Managed could support the development of positive elephant-keeper relationships and may buffer stress, thereby protecting against stereotypic behavior rate risk. Transferring elephants between zoos also has major social implications including separation from herdmates and known caretakers and introduction to unfamiliar elephants and humans. Social instability such as this is known to elicit a stress response and has been linked to stereotypic behavior performance in elephants [15] and other species [5]. Clearly these factors are multi-dimensional with several potential pathways for contributing to elephant stereotypy, but we think that the connection to sociality is particularly compelling given the highly social nature of elephants and their propensity to form strong social bonds [48].

It is also worth noting that the variables in our models were all developed from data collected at the individual elephant level. By contrast, zoo level variables (e.g., herd size, social group count, exhibit size, feeding/enrichment practices) failed to function well as predictors. In fact, it has been shown that zoo level data, particularly relating to housing and social factors, does not correlate well with individual level data due to the complexity and individualized nature of elephant management in contemporary zoos [17]. This is particularly important when housing and social variables are being collected for animal welfare assessment, because welfare outcomes such as stereotypic behavior are sensitive to differences in physical and social milieu. Therefore, it is our recommendation that future stereotypic behavior studies of zoo animals should not rely on zoo level data as a proxy for robust individual level variables. Additionally, our results indicate that follow up studies examining the relationship between stereotypic behavior and Percent Time In/Out Choice, Percent Time Managed, and Transfers could be highly valuable. These factors are newly discovered predictors of elephant stereotypic behavior and are associated with management practices that could potentially be modified by zoos. Longitudinal studies are also recommended, particularly with regards to understanding the long term effects of transfers and whether elephants transferred with familiar herdmates are protected from these effects.
